# Causal overstatements reduced in press releases following academic study of health news

**DOI:** 10.12688/wellcomeopenres.15647.2

**Published:** 2020-05-07

**Authors:** Luke Bratton, Rachel C. Adams, Aimée Challenger, Jacky Boivin, Lewis Bott, Christopher D. Chambers, Petroc Sumner

**Affiliations:** 1 School of Psychology, Cardiff University, Cardiff, Cardiff, CF10 3AT, UK; 2 Cardiff University Brain Research Imaging Centre (CUBRIC), School of Psychology, Cardiff University, Cardiff, UK

**Keywords:** science news, hype, exaggeration, science communication

## Abstract

**Background:** Exaggerations in health news were previously found to strongly associate with similar exaggerations in press releases. Moreover such exaggerations did not appear to attract more news. Here we assess whether press release practice changed after these reported findings; simply drawing attention to the issue may be insufficient for practical change, given the challenges of media environments.

**Methods:** We assessed whether rates of causal over-statement in press releases based on correlational data were lower following a widely publicised paper on the topic, compared to an equivalent baseline period in the preceding year.

**Results: **We found that over-statements in press releases were 28% (95% confidence interval = 16% to 45%) in 2014 and 13% (95% confidence interval = 6% to 25%) in 2015. A corresponding numerical reduction in exaggerations in news was not significant. The association between over-statements in news and press releases remained strong.

**Conclusions:** Press release over-statements were less frequent following publication of Sumner et al. (2014). However, this is correlational evidence and the reduction may be due to other factors or natural fluctuations.

## Introduction

News media help disseminate health information to millions of readers, but appealing news stories can contain misleading claims, with associated risks to public health ( Buhse *et al*., 2018; Grilli *et al*., 2002; Haneef *et al*., 2015; Matthews *et al*., 2016; Ramsay, 2013; Schwitzer, 2008; Sharma *et al*., 2003; Yavchitz *et al*., 2012).

Key sources of science and health news are press releases from journals, universities and funders ( Autzen, 2014; de Semir *et al*., 1998). Previous observational research has found that health news content is strongly associated with press release content, including when exaggerations occur ( Jackson & Moloney, 2016; Lewis *et al*., 2008; Schwartz *et al*., 2012; Sumner *et al*., 2014; Sumner *et al*., 2016; Yavchitz *et al*., 2012). 

However, it is not clear whether such research has much influence on the practice of academics and press officers in the preparation of press releases. Given the need to write short compelling statements about complex research, it is all too easy to inadvertently allow over-statements. We believe that the majority of exaggerations are not purposeful, but arise from the desire to be impactful, clear and accessible. It may be very difficult for this to change. The finding that many news exaggerations are already in press release text ( Sumner *et al*., 2014) certainly attracted interest and controversy ( BMJ Altmetric, 2019). It was received positively by many press officers who are motivated to communicate science carefully, and helped catalyse some initiatives ( The Academy of Medical Sciences, 2017). It was discussed in science communication conferences, blogs, twitter, and directly with press officer teams while developing a collaborative trial ( Adams *et al*., 2019).

However, we do not know if sharing awareness has any potential effect on practice. Here we simply assess whether the rate of over-stated claims was lower in the 6 months following publication of that article (January to June 2015) compared to the equivalent 6 months in the previous year (January to June 2014). Our main interest was press release claims – since these were the source identified in Sumner *et al*. (2014) – but we also assessed the associated news stories. Clearly our data can only establish whether a detectable difference occurred, and will not establish its cause. We can additionally compare the data to a third time-point in 2011 (with some limitations).

We focus here on causal claims based on correlational evidence, a common and potentially impactful form of over-statement in academia and science reporting ( Buhse *et al*., 2018; Ramsay, 2013; Wang *et al*., 2015). For example, Haber *et al*. (2018) found that in health news highly shared on social media, the causal language was over-stated relative to the underlying strength of the evidence in about half of the cases they studied. Meanwhile, Shaffer *et al*. (2018) provided some linking evidence that causal narratives in health news might genuinely affect readers’ health choices and intentions.

Distinguishing the types of evidence that can or cannot support causal inference is not intuitive ( Norris *et al*., 2003). The distinction is multifactorial, but at its heart is the difference between correlational (observational) and experimental evidence. That is not to say that all observational studies are unable to support a causal inference (if large, replicated and other factors are controlled). There is also the question of reliability and power for small samples. It is reported that around half the correlations underlying media causal claims are not confirmed by later meta-analyses ( Dumas-Mallet *et al*., 2017).

Sumner *et al*. (2014) tested three types of exaggeration: causal claims, advice, and human claims from animal experiments. The results for causal claims appear robust across several studies in different contexts or using different analysis protocols ( Adams *et al*., 2017; Adams *et al*., 2019; Bratton *et al*., 2019; Buhse *et al*., 2018; Schat *et al*., 2018; Sumner *et al*., 2014; Sumner *et al*., 2016). The results for exaggerated advice did not replicate in a subsequent sample ( Bratton *et al*., 2019), probably because exaggeration in advice is difficult to define in a way that applies to all cases. Human claims based on animal research have dropped in frequency in the UK since the Declaration on Openness on Animal Research (2012) and Concordat on Openness on Animal Research (2014), probably because one previous motivation for such ‘exaggerations’ was to avoid revealing animal research facilities previously ( Bratton *et al*., 2019; see also supplementary information in Adams *et al*., 2019). Therefore we focus here on causal claims based on correlational evidence evidence – which we will refer to as ‘causal over-statement’ – testing whether rates were lower in a six-month period in 2015 compared to the same months in 2014.

## Method

### Collection of press releases, journal articles and news

Press releases from 2014 and 2015 were collected from the same sample of 20 universities as used in Sumner *et al.* (2014), as well as from the BMJ, which published the paper. The press offices of these institutions were the most directly aware of the findings. This dataset is an expanded version of the dataset used in Bratton *et al*. (2019), which replicated Sumner *et al*. (2014) with the 2014 and 2015 samples from the 20 universities. The observation periods were January to June 2014 (pre-publication), and January to June 2015 (post-publication; Sumner *et al*., 2014 was published in December). We chose a 6-month period to aim for a sufficient number, and compared equivalent months in case press release output has seasonal changes (e.g. associated with academic year). Online repositories (websites, and EurekAlert.org) were searched for any press releases from the included institutions. This resulted in a corpus of 4706 press releases. The sample was then restricted to those relevant to human health, using the same criteria as ( Sumner *et al.*, 2014); which included all biomedical, lifestyle, public health and psychological topics), that reported on a single, published, peer-reviewed research article. This left 1033 relevant press releases. To ensure similar sample numbers across institutions and to reduce the sample to one we had resources to code, we implemented a cap of 10 press releases for each time period for each institution, through random selection where necessary. This resulted in a sample of 368 press releases, for which the associated peer-reviewed journal articles were retrieved. ** For each press release, relevant news articles (i.e. those which make reference to the source research) were collected via keyword searches using Google Search and the Nexis database (LexisNexis, New York, NY), up to 28 days after publication of the press release, and up to one week before (to allow for rare embargo breaches). The sample was then limited to cases where the study design was observational cross-sectional, observational longitudinal, or an observational meta-analysis (N=168 press releases). For analysing overstatements in press releases and news, we only used cases where the journal article was not already overstated (N=98 press releases; 322 news).

### Article coding

Prior to coding, the corpus of articles underwent a redaction process using Automator software (5.0, Apple Inc.) to remove any references to the year 2014 or 2015. This was so that the coders, who were aware of the aim of the study, were not aware which condition each article belonged to. The articles were coded using the standardised coding sheet used by Adams *et al*., 2017 (see raw data folder ‘before_after_data’ in ( Chambers *et al*., 2019)). For this analysis, only information regarding the statements of causal or correlational relationship was used. Two researchers (LB and AC or RCA) independently coded each article and any disagreements were discussed, with a third coder (AC or RCA) where necessary. This created a database with 100% agreement in coding.

*Coding of causal and correlational claims.* We used the scale developed by Adams *et al*. (2017), in which directly causal statements and *can cause* statements are classed as over-statements for correlational evidence. On the other hand, it was not classed as an over-statement if the claim contained *might, may, could, linked to, predicts, associated with* and other associative or conditional phrases. We refer to these phrases as ‘aligned’ with correlational evidence. Although Sumner *et al.* (2014) originally distinguished between some of these phrases, readers were found not to consistently rank any of them as stronger than the others ( Adams *et al*., 2017). In contrast, readers consistently ranked *can cause* and directly causal statements as stronger statements.

The strongest claims relating two variables in the study (e.g. a food and a disease) were recorded from the abstracts and discussion sections of journal articles. For press releases and news articles, the strongest statement was coded from the first two sentences of main text (where these were directly relevant to the research; general context was excluded).

We defined over-statements as causal or *can cause* claims based on correlational evidence. We only analysed cases where the journal article did not already make such claims, since our focus was not on claims taken straight from the publication, where publications lags mean that such causal claims may have been originally penned at any time in 2014 or early 2015. 

### Statistical analysis

Consistent with our previous approach ( Sumner *et al*., 2014), generalised estimating equations (GEE) were used (in SPSS version 24) to provide estimates and confidence intervals adjusting for the clustering of multiple articles to one source (multiple news articles from one press release; or multiple press releases from the same institution). The GEE is an extension of the quasi-likelihood approach and is used in situations in which data is clustered to estimate how much each data point should contribute statistically. The key part of the process is to estimate the correlation of data within clusters. At one extreme, all data within clusters might be fully correlated, in which case there is really only as many samples are there are clusters; separating the data points within clusters adds no additional information. At the other end of the extreme, data within clusters may be entirely uncorrelated; in this case the clustering does not matter and all data points can be treated as independent. In reality, data within clusters tends to be somewhat correlated, and the GEE estimates this and applies a weighting factor to those data points depending on the degree of correlation. The approach is accessibly explained by Hanley *et al.* (2003), so we do not replicate the equations here. We used a logit linking function because the data is binary, and an exchangeable working correlation, which is a common approach for clustered data and makes a parsimonious assumption that correlations are similar between all data within clusters.

## Results

### Press release overstatements

**Figure 1.**
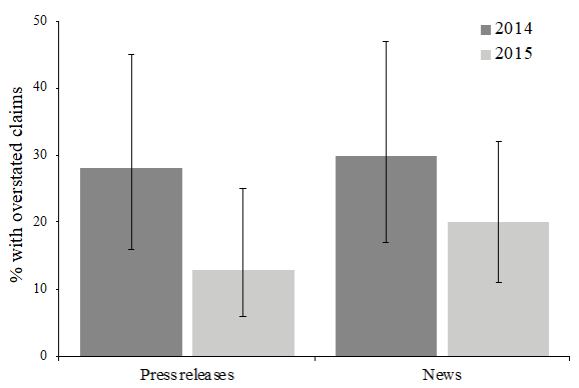
Overstatement rates in press releases and news in 2014 and 2015. The rate for press releases was significantly reduced in 2015 versus 2014. For news any apparent reduction was not significant. Error bars represent 95% confidence intervals.

In the sample from 2014, 28% (95% confidence interval = 16% to 45%) of press releases made a causal over-statement: a causal claim or *can cause* claim when the data were correlational and the journal article had not made a similar claim. In the sample from 2015, this rate was significantly lower, at 13% (95% confidence interval = 6% to 25%, see Figure 1). Thus, the odds of such overstatement were higher in 2014 (odds ratio = 2.7, 95% confidence interval = 1.03 to 6.97).

### Comparison to 2011

Adams *et al*. (2017) analysed a database of press releases from universities in 2011 using a definition of causal over-statement similar to the one used here. The number of press releases with causal over-statement was 19% (95% confidence interval = 14% to 25%). There were some methodological differences; it was a reananlysis of the data in Sumner *et al*. (2014), who collected a full year of press releases from 20 universities with no cap on numbers from each institution, and used partial instead of complete double coding.

### News overstatements

In the sample from 2014, 30% (95% confidence interval = 17% to 47%) of news made a causal overstatement. In the sample from 2015, this rate was 20% (95% confidence interval = 11% to 32%, see Figure 1). There was not a significant difference (95% confidence interval of the odds ratio = 0.6 to 4.8). These numbers can also be compared to those for news in 2011, analysed by Adams *et al*. (2017). The number of news with causal over-statement was 32% (95% confidence interval = 24% to 41%).

### News statements as a function of press release statements

**Figure 2.**
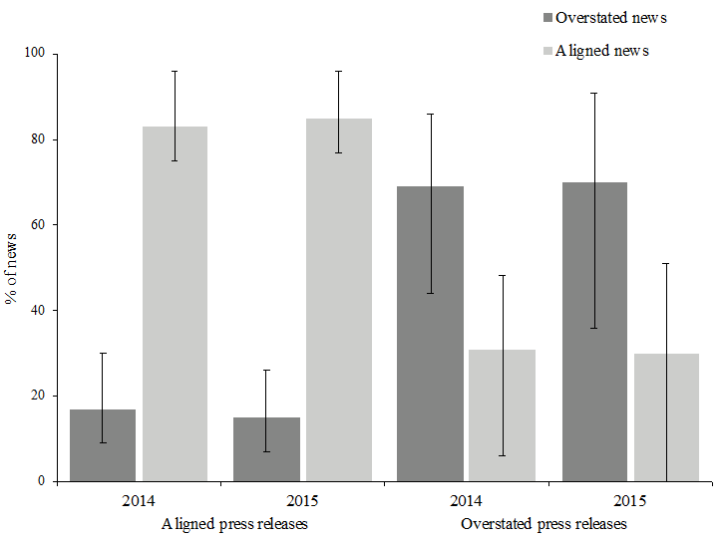
Causal overstatements in news articles as a function of press release overstatement and year of publication. ‘Aligned’ press releases or news are those that do not make causal claims stronger than *‘may cause’*. The association between news and press releases is present in both years and not statistically different between years. Error bars represent 95% confidence intervals.

To assess whether the drop in press release over-statements meant a weakening of the previously found association between news claims and press release claims, we assessed this association following the same methods as previously described ( Bratton *et al*., 2019; Schat *et al*., 2018; Sumner *et al*., 2014; Sumner *et al*., 2016). Across 2014 and 2015 combined, the odds of over-stated news claims were 12 times higher (95% confidence interval = 4.5 to 32) for over-stated press releases (69% news over-stated, 95% confidence interval = 49% to 84%), than for aligned press releases (16% news over-stated, 95% confidence interval = 10% to 24%). This association between news and press releases ( Figure 2) was not different between the years (odds ratio = 1.1, 95% confidence interval = .2 to 6.2) and is consistent with the association between news and press releases seen for exaggerations and other content previously.

## Discussion

We set out to assess whether there was evidence of changes in press release practice after academic publications about health news and press releases. We found an approximate halving (28% to 13%) in the rate of causal over-statements in press releases based on correlational evidence in the 6 months following a widely shared publication ( Sumner *et al*., 2014) compared to an equivalent 6 months the preceding year ( Figure 1). These rates can additionally be compared to the rate of 19% in a dataset from 2011 (abeit with some differences in methodology).

This evidence is correlational itself, and may not mean that the publication caused the change, since other factors may also have changed between 2014 and 2015. There has been scrutiny of health news and press releases from multiple quarters, and also press officer staff turnover may spontaneously change the balance of language in causal claims. At one extreme, it is possible that the changes were fully random; that 2014 was an unusually high year, and a drop to 13% was merely natural fluctuation/regression to the mean. At the other extreme is a fully causal explanation; that press releases were on a trajectory of rising causal over-statement, and awareness-raising reversed that trend. The truth normally lies somewhere between extreme interpretations, and all the above factors may have played a role. Moreover, whatever the causal chain, the drop or fluctuation shows that a high rate of causal language is not inevitable in press releases, despite the need to be concise and appealing.

Beyond the main focus on press releases, we also saw a numerical reduction in overstatements in news, but this was not significant ( Figure 1). However, we found a strong association between news and press release language ( Figure 2), consistent across years and consistent with previous research ( Bratton *et al*., 2019; Schat *et al*., 2018; Sumner *et al*., 2014; Sumner *et al*., 2016). There was no reason for this association to change while the time pressures on journalists remain intense, and importantly it did not weaken with the reduction of overstatement in press releases. This strong association raises the question of why significant reduction in press release overstatement was not mirrored in significant reduction in news overstatement ( Figure 1), if a causal chain were operating such that press release claims influence news claims.

In fact the data are consistent with such a causal effect, because it is expected to be diluted by other factors. Numerically, if news carries overstatements for around 70% of overstated press releases and 15% of non-overstated press releases (e.g. Figure 2), and if this is difference is causal, we can calculate the expected change in news overstatement as a result of the change in press release overstatement seen in Figure 1 (28% to 13%). We would therefore expect overstatements in around 30% (0.7*28+0.15*72) of news in 2014 and 22% (0.7*13+0.15*87) of news in 2015, which is close to what we saw, and clearly a diluted effect compared with the press release reduction of 28% to 13% (this outline calculation assumes similar news uptake for press releases regardless of overstatement, consistent with previous results; ( Bratton *et al*., 2019; Schat *et al*., 2018; Sumner *et al*., 2014; Sumner *et al*., 2016). Therefore our results are consistent with the non-significant effect in news being due to dilution and insufficient power, and should not be taken as evidence for no change in the news despite a difference in press releases.

We based our analysis on press releases and news for journal articles that did not already make causal claims. Of additional note, a GEE analysis of the journal articles themselves showed there were already causal claims in an estimated 40% of the 168 peer-reviewed journal articles based on correlational evidence (and meeting our other inclusion criteria). This tendency to use causal language, even in peer reviewed research conclusions, has been noted previously ( Wang *et al*., 2015). It would be worth following such rates to find if they too might show signs of decreasing as awareness is raised.

We analysed only one form of overstatement – causal claims, which are a cornerstone of scientific inference. There are many other forms of potential overstatement that we did not analyse, including the two originally assessed by Sumner *et al*. (2014): advice to readers and human inference based on non-human research. There are different reasons why we did not use these here as a test for professional practice change. For advice, we cannot compare it objectively to an aspect of study methods, and have recently reported that the association between advice in news and press releases did not replicate (using a subset of the 2014/15 sample used here; Bratton *et al*., 2019). We believe this may show that advice exaggeration is difficult to define. Audiences change between journal articles, press releases and news, and thus the appropriate phrasing of advice may legitimately change. For human inference from non-human research, the publication date of Sumner *et al.*, 2014 was confounded with the Concordat on Openness on Animal Research in the UK, which was signed by the majority of the institutions in this replication from May 2014 onwards. This is likely to be the major driver of drops in the rate of animal research studies being described in human terms ( Adams *et al*., 2019; Bratton *et al*., 2019).

## Conclusions

Previous converging evidence suggests that press releases have a strong influence on health and science news, which in turn influences public health decisions and health practioners (see *Introduction*). Here, we found a reduction in press release causal overstatement associated with the publication of a study examining news and press release exaggerations. Although correlational, this evidence may suggest that press release practice can change in response to research, given that casual overstatements do not seem to have been decreasing prior to this. However, natural fluctuation or other factors that changed between 2014 and 2015 could also explain the data.

## Data availability

### Underlying data

Open Science Framework: ISRCTN10492618: RCT of optimal press release wording on health-related news coverage, https://doi.org/10.17605/OSF.IO/APC6D ( Chambers *et al*., 2019). This version of the project was registered on 14 ^th^ December 2019: https://osf.io/y7t3p.

The underlying data used for this study can be found in folder ‘Processed data’, subfolder ‘ Impact assessment for Sumner *et al.*’.

Data are available under the terms of the Creative Commons Attribution 4.0 International license (CC-BY 4.0).
