# Supplementary material for: Causal overstatements reduced in press releases following academic study of health news
Source: Wellcome Open Res. 2020 May 7;5:6. Originally published 2020 Jan 8. [Version 2] doi: 10.12688/wellcomeopenres.15647.2 (PMC7236584; doi:10.12688/wellcomeopenres.15647.2)
Supplement: Peer review history

# Peer review: Reviewer response for version 2

**DOI:** 10.21956/wellcomeopenres.17456.r38679

**Published:** 2020-05-18

**Authors:** Alexandra L. J. Freeman

**Affiliations:** 1 Winton Centre for Risk and Evidence Communication, University of Cambridge, Cambridge, UK

The authors have addressed all my points in this new version, and I am very happy to recommend indexing.

Is the work clearly and accurately presented and does it cite the current literature?

Yes

If applicable, is the statistical analysis and its interpretation appropriate?

I cannot comment. A qualified statistician is required.

Are all the source data underlying the results available to ensure full reproducibility?

Yes

Is the study design appropriate and is the work technically sound?

Yes

Are the conclusions drawn adequately supported by the results?

Partly

Are sufficient details of methods and analysis provided to allow replication by others?

Yes

Reviewer Expertise:

Evidence communication.

I confirm that I have read this submission and believe that I have an appropriate level of expertise to confirm that it is of an acceptable scientific standard.

# Peer review: Reviewer response for version 2

**DOI:** 10.21956/wellcomeopenres.17456.r38678

**Published:** 2020-05-13

**Authors:** Kristian H. Nielsen

**Affiliations:** 1 Centre for Science Studies, Aarhus University, Aarhus, Denmark

Thanks to the authors for responding positively and constructively to my previous comments. I am happy with the revisions they have made.

Is the work clearly and accurately presented and does it cite the current literature?

Yes

If applicable, is the statistical analysis and its interpretation appropriate?

I cannot comment. A qualified statistician is required.

Are all the source data underlying the results available to ensure full reproducibility?

Yes

Is the study design appropriate and is the work technically sound?

Yes

Are the conclusions drawn adequately supported by the results?

Partly

Are sufficient details of methods and analysis provided to allow replication by others?

Yes

Reviewer Expertise:

Science communication.

I confirm that I have read this submission and believe that I have an appropriate level of expertise to confirm that it is of an acceptable scientific standard.

# Peer review: Reviewer response for version 1

**DOI:** 10.21956/wellcomeopenres.17144.r37829

**Published:** 2020-03-06

**Authors:** Kristian H. Nielsen

**Affiliations:** 1 Centre for Science Studies, Aarhus University, Aarhus, Denmark

Health communication is a complex evolving system with multiple agents employing a wide variety of communication channels. Important nodes in the system are research institutions and the news media. Researching into the relation between research and the media remains key to understanding the ways in which the system operates. Trustworthiness is particularly important to research institutions. If the information provided by research cannot be trusted – for example if the information turns out to be unreliable, inaccurate, or exaggerated – then the system “niche” for research might lose its relevance.

 This paper follows up on studies of what Alexandra L.J. Freeman in her review calls “causal over-statement” or “unwarranted/unsupported causal claims”. Previous studies by the same team and others have found correlation between unwarranted causal claims in press releases from universities and in related news stories (unwarranted, because they were based on statistical correlations and not on actual cause-effect relations). The team’s first study came out in December 2014, reporting on press releases from 20 leading British universities.

 The present study looks at press releases and media reports from the first half of 2014 (before the first study made news) and from the first half of 2015 (just after). There is a higher number of unwarranted causal claims in the 2014 press releases (28%) compared to the 2015 ones (13%). The difference is statistically significant. However, there is not a significant difference between the number of unwarranted causal claims in the 2014 media reports compared to the 2015 ones.

 At the same time, the study finds “strong association” between “aligned” press releases and “aligned” news stories and “overstated” press releases and “overstated” news stories. (“Aligned” is not defined in the present nor in the original study, but probably means something like no causal claims based on correlational evidence.) This association, the authors note, raises the question of why the found drop in unwarranted causal claims in the press releases is not matched by a similar drop in unwarranted causal claims in the news stories. They speculate that “the effect on news is diluted by other factors and so here we may have had sufficient power only to detect the effect on press releases”.

 It is not entirely clear what this sentence implicates in terms of cause and effect, but probably the effect on news caused by the original study and mediated by the study’s effect on press releases. It is unfortunate that the authors discuss their findings in terms of cause and effect when what they really have is correlational evidence, particularly in a paper that deals with unwarranted causal claims.

 Nevertheless, the findings are interesting and generally clearly reported. I support Freeman’s suggestion to replace the term “causal claims about correlation” with another term. As Freeman also points out in her review, the results obviously needs to be taken with some reservation due to the limited number of data points (just two). In my view, the study’s most important implication is that it opens up for analyzing the potential impact of academic research on health communication in conjunction with press releases and news stories.

 In order for the reader to understand the reception of the original study, it would have been nice with a little more background in the introduction. The paper says that the original study “attracted interest and controversy” with a reference to BMJ Altmetric. I encourage the authors to expand on the context in which this study was carried out and provide a full overview of the research activities in which this study is embedded.

Is the work clearly and accurately presented and does it cite the current literature?

Yes

If applicable, is the statistical analysis and its interpretation appropriate?

I cannot comment. A qualified statistician is required.

Are all the source data underlying the results available to ensure full reproducibility?

Yes

Is the study design appropriate and is the work technically sound?

Yes

Are the conclusions drawn adequately supported by the results?

Partly

Are sufficient details of methods and analysis provided to allow replication by others?

Yes

Reviewer Expertise:

Science communication.

I confirm that I have read this submission and believe that I have an appropriate level of expertise to confirm that it is of an acceptable scientific standard.

# Response

**Published:** 2020-04-27

**Authors:** Petroc Sumner

**Affiliations:** Cardiff University, UK

Thank you for your very helpful comments. We now defined ‘aligned’ in methods and the legend of figure 2. We have rephrased and expanded the sentence ‘the effect on news is diluted by other factors and so here we may have had sufficient power only to detect the effect on press releases’. As the reviewer notes, it is very difficult to avoid causal language even when we are primed to do so by the very topic of the paper. It is unfortunate that our statistical terms do not differentiate between an ‘effect’ in the data that is merely a correlation and a causal ‘effect’. The effect we refer to here is the hypothetical effect press release text has on news text – consistent with the strong correlation between news and press release statements seen in several studies (and again in Figure 2 of the current paper). We now note more explicitly that the causal effect is hypothetical, and the data is merely consistent with this. We have also adopted the phrase ‘causal over-statement’, as recommended, and added more information about the reception of the original study in the introduction.

# Peer review: Reviewer response for version 1

**DOI:** 10.21956/wellcomeopenres.17144.r37827

**Published:** 2020-02-17

**Authors:** Alexandra L. J. Freeman

**Affiliations:** 1 Winton Centre for Risk and Evidence Communication, University of Cambridge, Cambridge, UK

The evidence pipeline in healthcare from academic research to public news reporting and public behaviour is a leaky one. Incentive structures, interests and resource pressures at each stage lead to a form of 'Chinese whispers' that means that the public rarely end up being able to align their health behaviours appropriately to the best research evidence.

 In 2014, Sumner *et al.* ^1^ investigated one part of the chain: the link between what is reported in news articles and headlines and in the press releases that likely triggered those reports. They showed that a lot of 'exaggerated claims' in the news were actually already present in the press releases on which those reports were based.

 After publicising these findings and calling for greater awareness of the responsibilities of press officers in minimising exaggeration in the media, this new study by the team seeks to look for any potential change in behaviour by press officers. They do this by comparing the levels of 'exaggerated claims' both before and after the publication of their previous findings.

 In order to look for a potential change in the number of exaggerated claims, the team compare the number of unwarranted causal claims in news reports and in press releases over a 6 month period before publication of their previous paper (in 2014) and after (in 2015). They find a significant decrease in unwarranted causal claims in press releases, and a smaller decline (not statistically significant) in news articles.

 I will leave it to others to critique the statistical methods used and check the claimed findings against the raw data (which are available and well-annotated), as I do not have the relevant experience.

 The fact that unwarranted causal claims have decreased by a considerable degree is an interesting (and encouraging) finding. Whether it can really be attributed to publication of their 2014 paper is uncertain. The authors freely admit that this is a correlation, although claims that this indicates that 'press release practice is malleable' seem a little strong.

 The team have only two data points: 2014 and 2015, and there are a lot of potential confounders. They themselves point out that they were unable to do a similar analysis of claims of findings based on animal research being of relevance to humans, because of the confounding influence of the Concordat on Openness on Animal Research in 2014. There may well be other confounding influences on the claims of unwarranted causality which they report here. The authors do acknowledge this in the Discussion, but discount other factors as being unlikely to cause such a large decrease.

 The team also mention that 'there appears to have been a rise between 2011 and 2014'. The apparent fall in 2015 could therefore have included a degree of regression to the mean after a particularly heinous year for exaggeration in 2014. Adams *et al.* (2017) ^2^ refer to data from 2011 and 2014, meaning that this present study had three data points which it could have presented (2011, 2014 & 2015), rather than just two (2014 and 2015). Was the level of unwarranted causal claims in 2011 comparable to 2015? This would have weakened their argument for the publication in 2014 being responsible for observed fall between 2014 and 2015, but should be disclosed to allow readers to judge for themselves.

 In summary, then, I think that these findings are interesting and seem methodologically sound, but I would not draw too much conclusion from a single pair of data points (especially when a third is hinted at that might undermine the proposed pattern). Stronger evidence would have been a longer time series, preferably mixed with qualitative research with press officers about their awareness of the Sumner (2014) publication, and perhaps a study comparing the practices of institutions who had and had not been exposed to the findings of Sumner (2014). However, I recognise that this article is the product of additional analysis of a dataset collected for other purposes and that it does not stand alone in the literature. I would therefore advise that the conclusions are not couched too strongly, and that the conclusions are framed to be based on the totality of the evidence rather than only these results.

 A few minor points:

- In the title and throughout the manuscript, the phrase 'causal claims about correlations' is a little hard to read. Using the shorter 'causal over-statement' or 'unwarranted/unsupported causal claims' in places might make it easier to read. This is particularly a problem because the manuscript itself is reporting a correlation and so the terminology can get complex.
- I was surprised that the article didn't reference Haber *et al. (*2019) ^3^, which also reviewed causal language in news reports and in the original articles in a dataset from 2015. Although they are addressing a slightly different aspect of the evidence chain, their findings are probably worth drawing attention to where the authors currently cite Wang *et al.* (2015). (Of less relevance to this article specifically, but Shaffer *et al.* (2018) ^4^ give evidence on the link between news reporting and health behaviour intentions in readers which nearly completes the chain).
- I think there's a typo of '2014' for '2015' (as well as a misspelling of 'overstatements' in the sentence "we would expect aoverstatements in around 30% (0.7*28+0.15*72) of news in 2014 and 22%(0.7*13+0.15*87) of news in 2014".

Is the work clearly and accurately presented and does it cite the current literature?

Yes

If applicable, is the statistical analysis and its interpretation appropriate?

I cannot comment. A qualified statistician is required.

Are all the source data underlying the results available to ensure full reproducibility?

Yes

Is the study design appropriate and is the work technically sound?

Yes

Are the conclusions drawn adequately supported by the results?

Partly

Are sufficient details of methods and analysis provided to allow replication by others?

Yes

Reviewer Expertise:

Evidence communication.

I confirm that I have read this submission and believe that I have an appropriate level of expertise to confirm that it is of an acceptable scientific standard, however I have significant reservations, as outlined above.

## References

[1] : The association between exaggeration in health related science news and academic press releases: retrospective observational study. *BMJ*.2014;349: 10.1136/bmj.g7015 g7015 10.1136/bmj.g7015 25498121PMC4262123

[2] : How readers understand causal and correlational expressions used in news headlines. *J Exp Psychol Appl*.23(1) : 10.1037/xap0000100 1-14 10.1037/xap0000100 27808530

[3] : Causal language and strength of inference in academic and media articles shared in social media (CLAIMS): A systematic review. *PLoS One*.2018;13(5) : 10.1371/journal.pone.0196346 e0196346 10.1371/journal.pone.0196346 29847549PMC5976147

[4] : What Is the Story with Narratives? How Using Narratives in Journalism Changes Health Behavior. *Health Commun*.33(9) : 10.1080/10410236.2017.1333562 1151-1157 10.1080/10410236.2017.1333562 28657356

# Response

**Published:** 2020-04-27

**Authors:** Petroc Sumner

**Affiliations:** Cardiff University, UK

Thank you for your very helpful comments. We have added more information about the data from 2011 to enable a better comparison. We have noted in abstract and discussion that regression the mean / natural fluctuation is a possible explanation, and removed the phrase 'press release practice is malleable'. We have also adopted the phrase ‘causal over-statement’, as recommended. We have included Haber et al. and Shaffer et al. where recommended. We have corrected the typos.
